# Clinical characteristics of children with juvenile dermatomyositis recruited within the first 7 months of the CARRAnet registry

**DOI:** 10.1186/1546-0096-10-S1-A12

**Published:** 2012-07-13

**Authors:** Mark F Hoeltzel, Mara L Becker, Angela B Robinson, Brian M Feldman, Adam Huber, Ann M Reed

**Affiliations:** 1 Children's Mercy Hospital, Kansas City, MO, USA; 2 IWK Health Centre, Halifax, NS, Canada; 3 Mayo Clinic, Rochester, MN, USA; 4 Rainbow Babies and Childrens Hospital, Cleveland, OH, USA; 5 The Hospital for Sick Children, Toronto, ON, Canada; 6 CARRA, Stanford, CA, USA

## Purpose

Performing quality clinical and translational research in juvenile dermatomyositis (JDM) is difficult due to the rarity of the disease. The Childhood Arthritis and Rheumatology Research Alliance (CARRA) initiated a multi-center observational cohort study to create a foundational clinical database for the major rheumatic diseases of childhood, including JDM. Initial data from the JDM cohort (prevalent and incident cases) enrolled in the first 7 months of this ongoing study are described here.

## Methods

Children under the age of 21 with onset of JDM prior to 16 yrs old were included. JDM was established using modified Bohan and Peter criteria. Subjects or their guardians were consented, and clinical data were collected from the patients/guardians and medical providers using both general and JDM-specific case report forms at the time of enrollment. Data regarding demographics, disease characteristics, diagnostic assessment, and medication exposure were collected. Baseline measures of muscle strength, physical functioning, and quality of life were performed, including the Childhood Myositis Assessment Scale (CMAS), Childhood Health Assessment Questionnaire (CHAQ), Health Related Quality of Life measure (HRQOL), ACR Functional Class rating, global disease assessments, and pain scores. Data were pooled and stored in a secure centralized database and de-identified prior to analysis. IRB approval was obtained at each enrolling site.

## Results

**Figure 1:**
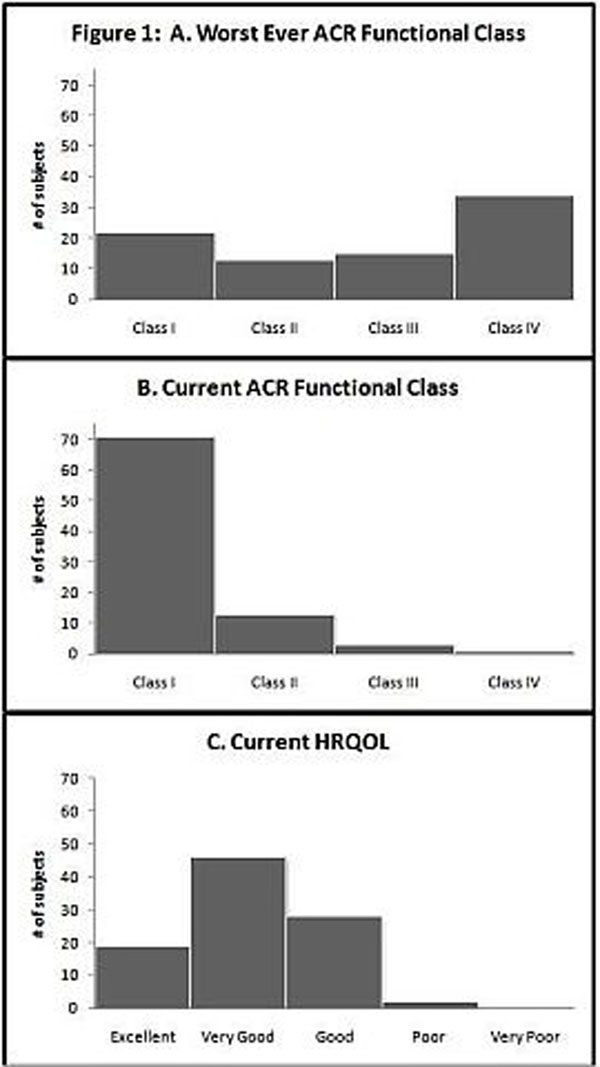


Between May 28, 2010 and December 28, 2010, 102 subjects meeting modified Bohan and Peter criteria for JDM were enrolled from 23 sites in the U.S. The average number of patients enrolled per site was 5.3 (range 1-18). A summary of subject demographics and disease characteristics is provided in Table 1. Median (quartiles) CMAS score at enrollment was 50 (45, 52) with a range from 0-52. Median CHAQ score at enrollment was 0 (0, 0.5) with a range from 0-3. Median physician and subject global assessment scores at enrollment were 1 (0, 2) with a range from 0-8, and 1 (0, 4) with a range from 0-9 respectively. Subject-reported median pain score at enrollment was 1 (0, 2) with a range from 0-8. HRQOL and ACR functional class results are represented in Figure 1.

**Table 1:** Demographics and disease characteristics

Characteristc	Number (%) of subjects		Characteristic	Number (%) with characteristic *ever*	Number (%) with characteristic *at enrollment*
Sex			Elevated muscle enzymes	86/89 (97%)	6/89 (7%)
Female	80 (78%)		Arthritis	34/86 (40%)	4/86 (5%)
Male	22 (22%)		Calcinosis	10/87 (12%)	7/87 (8%)
Ratio Female:male	3.6:1		Cardiac involvement	1/85 (1%)	1/85 (1%)
Race/Ethnicity			GI Ulceration	3/85 (4%)	1/85 (1%)
White, Non-Hispanic	65 (64%)		Dysphagia/Dysphonia	26/84 (31%)	1/84 (1%)
White, Hispanic	13 (13%)		ILD	1/84 (1%)	0/84 (0%)
African American	9 (9%)		Muscle weakness:		
American Indian	1 (1%)		None	-	62/91 (68%)
Pacific Islander	1 (1%)		Mild	-	23/91 (25%)
Multi-racial	10 (10%)		Moderate	-	4/91 (4%)
Unknown	3 (3%)		Severe	-	2/91 (2%)
1° Family hx of autoimmunity	21/92 (23%)		Periungual telangiectasia	-	39/86 (45%)
Positive ANA	55/85 (65%)		Contractures	-	7/91 (8%)
			
**Chronology**	**Median, yrs (quartiles)**	**Range, yrs**	V or shawl sign	-	5/91 (6%)
			
Age	10.6 (7.1, 14.8)	2.4-20.8	Lipodystrophy	-	4/91 (4%)
Age of onset	6.0 (3.3, 9.5)	0.9-15.9	Skin ulcer	-	3/91 (3%)
Disease duration	3.0 (1.7, 5.7)	0.2-12.8	Malar or facial erythema	-	24/90 (27%)
Time to rhematologie care	0.32 (0.13, 0.67)	0-5.1	Gottron sign, papules, or heliotrope	-	44/91 (48%)

## Conclusion

In 7 months, the ongoing CARRA registry of children with rheumatic diseases has collected clinical data on 102 children with JDM and has the potential to become one of the largest JDM cohorts in the world. This registry provides the infrastructure needed to advance clinical and translational research and represents a major step towards improving outcomes of children with JDM.

## Disclosure

Mark F. Hoeltzel: None; Mara L. Becker: None; Angela B. Robinson: None; Brian M. Feldman: None; Adam Huber: None; Ann M. Reed: None; Juvenile Myositis CARRA Subgroup: None; CARRAnet Investigators: None.

